# Adaptive RGB-D Semantic Segmentation with Skip-Connection Fusion for Indoor Staircase and Elevator Localization

**DOI:** 10.3390/jimaging11080258

**Published:** 2025-08-04

**Authors:** Zihan Zhu, Henghong Lin, Anastasia Ioannou, Tao Wang

**Affiliations:** 1 Fujian Provincial Key Laboratory of Information Processing and Intelligent Control, School of Computer and Data Science, Minjiang University, Fuzhou 350108, China; 2 Department of Computer Science and Engineering, European University Cyprus, Nicosia 1516, Cyprus

**Keywords:** RGB-D, semantic segmentation, skip-connection fusion, staircase and elevator localization

## Abstract

Accurate semantic segmentation of indoor architectural elements, such as staircases and elevators, is critical for safe and efficient robotic navigation, particularly in complex multi-floor environments. Traditional fusion methods struggle with occlusions, reflections, and low-contrast regions. In this paper, we propose a novel feature fusion module, Skip-Connection Fusion (SCF), that dynamically integrates RGB (Red, Green, Blue) and depth features through an adaptive weighting mechanism and skip-connection integration. This approach enables the model to selectively emphasize informative regions while suppressing noise, effectively addressing challenging conditions such as partially blocked staircases, glossy elevator doors, and dimly lit stair edges, which improves obstacle detection and supports reliable human–robot interaction in complex environments. Extensive experiments on a newly collected dataset demonstrate that SCF consistently outperforms state-of-the-art methods, including PSPNet and DeepLabv3, in both overall mIoU (mean Intersection over Union) and challenging-case performance. Specifically, our SCF module improves segmentation accuracy by 5.23% in the top 10% of challenging samples, highlighting its robustness in real-world conditions. Furthermore, we conduct a sensitivity analysis on the learnable weights, demonstrating their impact on segmentation quality across varying scene complexities. Our work provides a strong foundation for real-world applications in autonomous navigation, assistive robotics, and smart surveillance.

## 1. Introduction

Deep learning has revolutionized computer vision, particularly in semantic segmentation, yet existing methods struggle with accurately distinguishing architectural structures such as stairs and elevators under diverse conditions. Despite advancements, segmentation models often misclassify these critical elements, leading to navigation failures, safety risks, and accessibility barriers in real-world robotic applications. This gap is especially evident in assistive robotics for individuals with mobility impairments, autonomous navigation in public spaces, and robotic systems deployed in complex architectural environments. Precise recognition of stairs and elevators is essential for ensuring safe traversal, preventing missteps, and enabling seamless floor-to-floor navigation. Robust segmentation not only enhances obstacle detection and scene understanding but also plays a vital role in securing reliable robot performance against varying illumination, occlusion, and depth perception challenges.

However, traditional segmentation models often struggle to effectively integrate multimodal data, such as RGB and depth information, which limits their ability to capture complementary features. RGB data provides rich color and texture information, while depth maps capture geometric and structural cues. The inability to effectively fuse these two modalities can lead to suboptimal segmentation results, particularly in environments with low lighting, reflective surfaces, or complex architectural layouts.

To address this limitation, we propose Skip-Connection Fusion (SCF), a novel feature integration approach, by introducing a custom module before the decoder stage of the segmentation pipeline. This module leverages a concatenation-based method to fuse features extracted from RGB images and depth maps. Unlike conventional methods, our module employs learnable parameters to dynamically weight and distribute fused features, enhancing the network’s ability to discern and adapt to scene-specific characteristics.

By integrating this module into the segmentation framework, we aim to improve the robot’s ability to recognize and segment complex structures such as stairs, elevator doors, and interiors. The enhanced feature fusion process ensures that the depth information complements the texture and color features of the RGB images, leading to more accurate and robust segmentation results, especially in low-contrast and occluded environments.

This paper presents the experimental validation of our method, demonstrating its superior performance compared to existing models such as PSPNet [1] and Deeplabv3 [2]. Through detailed analyses, we illustrate how the proposed module enhances segmentation quality, providing a practical solution for robots operating in diverse and intricate environments.

To provide a broader context for our work, we refer readers to several recent reviews on semantic segmentation and its applications in challenging environments. For comprehensive discussions on deep learning-based semantic segmentation methods for low-contrast images, see [3]. A detailed review focusing on 3D semantic segmentation techniques is presented by [4], while a thorough survey on semantic segmentation for indoor scenes can be found in [5]. These studies offer valuable insights into the state of the art and ongoing challenges in the field.

### 1.1. Limitations

Despite significant advancements, existing methods still fail to address the unique challenges of architectural segmentation, particularly for complex structures such as stairs and elevators. Current approaches are generally designed for broad scenarios and lack the specialized mechanisms required to handle intricate geometries, variable lighting, and reflective surfaces typical in architectural environments:

- **Reflective and Translucent Surfaces:** Current models struggle with insufficient geometric cues, leading to inaccurate segmentation of reflective elevator doors.
- **Ineffective Fusion of Complementary Modalities:** Current approaches rely largely on generic fusion strategies that do not specifically cater to the unique demands of architectural segmentation. In particular, these methods often fail to dynamically integrate RGB and depth information, resulting in suboptimal feature fusion in complex environments.

### 1.2. Key Contributions

To address the limitations of existing segmentation methods, we propose a novel framework that integrates several innovative components:

- **Learnable Skip-Connection Fusion (SCF):** A dynamic fusion module that adaptively balances RGB and depth features through learnable weighting mechanisms. This design enhances segmentation robustness by effectively handling reflective and translucent surfaces commonly encountered in architectural environments.
- **Integration with PSPNet:** Our framework leverages pyramid pooling within the PSPNet architecture to capture both global and local contextual information. This integration significantly improves segmentation accuracy, particularly in cluttered and intricate scenes.
- **Task-Specific Optimization:** The proposed method is specifically tailored to segment stairs and elevators, achieving a favorable trade-off between high segmentation accuracy and computational efficiency in real time.
- **Dataset Contribution:** We have collected a dedicated dataset comprising 2386 images from real-world environments at Minjiang University in Fuzhou, Fujian Province, China. The dataset includes 1818 staircase images and 568 elevator images, captured from two camera perspectives to simulate robotic vision. The segmentation task is defined over six classes: upstairs, downstairs, elevator interior, elevator door, elevator frame, and background. All images were pre-processed via segmentation and padding and resized to 640 × 480 pixels, with 1671 images used for training and 715 for validation.
- **Problem Significance:** We address a critical yet relatively overlooked challenge in indoor robotic navigation—precise segmentation of stairs and elevators. Accurate recognition of these architectural structures is essential for ensuring safe and efficient navigation in multi-floor environments. Misclassification can lead to serious consequences, such as navigation failures, accessibility issues, and potential safety hazards for both robots and humans. Our approach introduces a novel fusion mechanism that improves segmentation accuracy, making autonomous navigation more reliable in real-world scenarios.
- **Methodological Contribution:** We propose a novel Skip-Connection Fusion (SCF) module that dynamically integrates RGB and depth features for enhanced segmentation. Unlike conventional concatenation-based fusion, SCF learns adaptive weights to balance modality contributions, effectively mitigating challenges such as occlusions, reflections, and variations in lighting conditions. Using this approach, we significantly improve the segmentation performance of robotic perception systems in complex indoor environments.

By combining these innovations, our framework significantly enhances segmentation performance in real-world architectural scenarios, addressing the unique challenges posed by these environments. Our SCF module builds on these advancements by introducing adaptive weighting for RGB and depth features. This approach dynamically prioritizes mode-specific advantages, addresses the identification of stairs and elevators for refined classification, and effectively deals with the limitations of reflective or translucent surfaces.

This paper is organized as follows. In Section 2, we review the related work in the field of semantic segmentation and its challenges, particularly in complex environments. Section 3 describes the methodology, including the proposed Skip-Connection Fusion (SCF) module and its integration into the segmentation pipeline. Section 4 presents the experimental evaluation of our approach, comparing it with state-of-the-art methods on a newly collected dataset. Finally, Section 5 concludes this paper and discusses potential directions for future research.

## 2. Related Work

### 2.1. Multimodal Fusion for Semantic Segmentation

Significant advances in semantic segmentation have been achieved through innovations in dilated convolution and conditional random fields, including the Fully Convolutional Network [6], PSPNet, DeepLab, SETR, and Segmentor series of methods, which have substantially improved segmentation performance. Building on these methods, multimodal data fusion, particularly integrating RGB and depth modalities, has emerged as a transformative approach that addresses the limitations of single-modality systems. This fusion is especially valuable in complex architectural spaces like stairs and elevators, where depth cues enhance geometric delineation, enabling models to handle challenges such as occlusions, reflective surfaces, and varying lighting conditions more effectively.

#### 2.1.1. Early and Foundational Approaches

Gupta et al. (2014) [7] laid the groundwork for RGB-D fusion by introducing HHA encoding, a method that converts depth data into three channels representing horizontal disparity, height above ground, and the angle between the pixel’s surface normal and gravity direction. This encoding aligned depth information with RGB features, enhancing segmentation accuracy by exploiting geometric and structural details. However, their reliance on direct concatenation of features limited their framework’s adaptability in handling reflective or translucent surfaces, which are often present in architectural environments.

Building on these foundational ideas, Lagos et al. (2022) [8] proposed the SemSegDepth model, which incorporates the concept of semantic objectness to better capture geometric relationships between segmentation and depth completion tasks. Their model employs a ResNet-50 backbone, a Feature Pyramid Network, and a joint branch for combined semantic and depth feature learning. While effective for improving segmentation accuracy, their approach struggled in scenarios lacking distinct geometric cues, such as untextured or flat surfaces.

These limitations highlighted the need for more adaptive feature fusion techniques, inspiring advancements like our Skip-Connection Fusion module, which enhances depth interaction by learning adaptive weights for feature blending. By addressing the challenges of previous methods, our module ensures robust segmentation even in environments with ambiguous or minimal geometric cues.

#### 2.1.2. Advanced Fusion Techniques: Feature-Level Integration

Recent studies have increasingly focused on feature-level integration to fully exploit the complementary strengths of RGB and depth modalities. Hazirbas et al. (2016) [9] proposed FuseNet, which introduced skip-connections for hierarchical feature fusion across modalities. This technique provided a robust baseline for multimodal integration and inspired the use of skip-connections in our fusion module to ensure effective feature propagation across scales.

More recently, the RDF-GAN framework (2022) [10] leveraged a GAN-based architecture incorporating W-AdaIN modules for fine-grained fusion of RGB and depth features. This method achieved state-of-the-art results in depth completion and segmentation by producing dense depth maps alongside confidence measures. However, its high computational cost posed challenges for real-time applications. Our method adapts this idea by using lightweight fusion layers, maintaining computational efficiency while enhancing segmentation accuracy in dynamic environments.

Zhang et al. (2023) [11] developed an attention-based fusion network tailored for RGB-D segmentation in cluttered indoor scenes. Their network dynamically weighted RGB and depth features, addressing occlusion and variable lighting conditions. While effective, their reliance on local attention mechanisms limited global context modeling. To overcome this, we integrate pyramid pooling layers in our framework, capturing global and local features to enhance segmentation in complex architectural spaces.

Additionally, Hao et al. (2024) [12] introduced PrimKD, a knowledge distillation-based approach that uses the RGB modality as a primary guide to enhance feature fusion. This principle aligns with our design philosophy of prioritizing the complementary strengths of RGB and depth data, as reflected in the dynamic weighting mechanisms of our fusion module.

Another significant contribution is MIPANet (2023) [13], proposed by Zhang and Xie, which utilizes a Multimodal Interaction Fusion Module and a Pooling Attention Module to harness interactive synergy between RGB and depth modalities. Their approach highlights the importance of feature-level interaction, which we further extend by introducing learnable parameters for adaptive fusion, enabling robust segmentation even in visually challenging architectural environments.

Finally, the diffusion-based framework employing deformable transformers for RGB-D semantic segmentation (2024) [14] demonstrated robustness in handling noisy measurements. This method’s focus on deformable feature integration directly informs our efforts to enhance segmentation reliability in cluttered or visually ambiguous scenarios, such as reflective elevator interiors or occluded staircases.

### 2.2. Segmentation of Architectural Structures

#### 2.2.1. Stair and Elevator Detection

Architectural elements like stairs and elevators present significant challenges in semantic segmentation due to their complex geometric structures and varying visual conditions. These challenges require precise boundary detection and robust multimodal fusion techniques to achieve accurate segmentation.

Wang et al. (2023) [15] introduced a stair detection network leveraging RGB and depth inputs via a selective fusion module, effectively extracting complementary features from both modalities. Their post-processing algorithms for geometric parameter estimation enabled real-time segmentation with enhanced accuracy, even under challenging lighting conditions. However, their method struggled with occlusions and reflective surfaces, which are prevalent in architectural environments. Building on their selective fusion principle, our method adopts a Skip-Connection Fusion (SCF) module designed to improve feature interaction between RGB and depth inputs. Unlike Wang et al., our SCF module integrates learnable parameters for dynamic weighting, allowing the model to adaptively prioritize features, thereby addressing challenges such as occlusions and reflective elements crucial for precise stair and elevator segmentation.

Our method addresses the gaps of the existing methods by integrating SCF with PSPNet, achieving both accuracy and computational efficiency for real-time deployment.

#### 2.2.2. Context-Aware Multi-Scale Fusion

Global and local context modeling is vital in complex architectural scenes, where features may be sparse, occluded, or highly variable. Jiang et al. (2022) [16] addressed this issue with a multi-scale framework, enhancing depth representation in cluttered environments by extracting hierarchical features. Inspired by their work, we employ multi-scale feature extraction within the PSPNet framework. Specifically, the pyramid pooling module captures global contextual cues, while our Skip-Connection Fusion (SCF) module dynamically integrates these cues with local RGB and depth features. This balanced fusion approach ensures robust segmentation performance in cluttered architectural scenarios, particularly for elements like stair edges and elevator interiors, where depth and texture information often overlap.

Kirch et al. (2023) [17] introduced a diffusion model conditioned on RGB data that excels in tasks requiring fine-grained depth estimation. However, their approach, optimized for humanoid-focused segmentation, lacked specificity for architectural elements such as stairs and elevators. We adapt the principle of conditional fusion from their work, embedding it within our SCF module. This adaptation ensures precise segmentation of geometric boundaries and transitions, addressing critical challenges such as occlusions and complex depth variations. Furthermore, leveraging PSPNet’s pyramid grouping to capture contextual information on multiple scales, our framework extends beyond stairs and elevators to other architectural features, such as door frames and hallways, where global and local signals are essential.

##### Extension to Other Architectural Features

While our approach primarily focuses on stairs and elevators, the combination of global–local context modeling via PSPNet and the SCF module is inherently generalizable. The ability to handle occlusion, cluttered environments, and complex geometric structures suggests potential applications to other architectural features, such as columns, arches, and intricate moldings. By leveraging the same principles of multimodal fusion and multi-scale feature integration, our method can provide robust segmentation solutions across a wide range of indoor and outdoor architectural elements.

##### Justification for Model Choices

While deeper networks like ResNet-101 provide improved accuracy, they increase computational overhead, making them less suitable for real-time applications. ResNet-50 offers a balance between accuracy and efficiency, making it ideal for embedded robotic applications. Similarly, the PSPNet framework addresses challenges such as occlusions and cluttered environments by using its pyramidal pooling module to extract global contextual information. This ensures that the model considers the broader spatial relationships in the scene, which is critical for distinguishing features in crowded or ambiguous settings. Complementarily, the SCF module enhances local feature integration by dynamically weighting RGB and depth inputs at multiple network layers. This mechanism allows the model to effectively delineate boundaries and transitions, particularly in regions with overlapping or reflective surfaces, such as stair railings and elevator doors. Alternative architectures like Swin-UNet and SegFormer were considered but introduce additional complexity that may not be justified for this task.

## 3. Methodology

In this section, we describe the SCF module in detail. Specifically, we first provide an overview of the training setup for PSPNet with ResNet-50 [18] and then introduce our feature fusion methods for RGB and depth images (The choice of ResNet-50 is for implementation consistency; our methodology remains valid with alternative backbone architectures).

### 3.1. Summary of SCF and Comparative Analysis

Existing RGB-D fusion methods face challenges in effectively distinguishing architectural structures like stairs and elevators under varying conditions, as shown in Table 1. FuseNet employs a dual-stream approach but lacks adaptability, making it sensitive to depth noise and less effective in complex scenes. RDF-GAN enhances feature representation using adversarial learning, but its high computational cost and unstable training process limit its practicality.

Our proposed Skip-Connection Fusion (SCF) module directly addresses these limitations by introducing a learnable fusion strategy that dynamically balances RGB and depth contributions. Unlike direct concatenation-based fusion, SCF emphasizes the structural characteristics of stairs and elevators, making it more robust in complex indoor environments. It effectively mitigates challenges such as illumination variations, occlusions, and reflections—critical factors for reliable segmentation in real-world applications. Additionally, integrating SCF with PSPNet enhances multi-scale context awareness, further improving segmentation accuracy across diverse lighting and viewpoint conditions. While SCF demonstrates superior adaptability, further evaluation across different building layouts and sensor types would provide deeper insights into its generalization capabilities.

### 3.2. Preliminaries

#### 3.2.1. Task Description

In supervised semantic segmentation, the objective is to map input images to their corresponding semantic labels using labeled datasets. The RGB dataset is defined as follows:

(1) $$D_{x}={\left\{\left(x_{i},y_{i}\right)\right\}}_{i=1}^{M_{x}},$$

where $x_{i}\in X$ represents the *i*-th RGB image, $y_{i}\in Y$ is its corresponding pixel-wise ground truth label, and $M_{x}$ is the total number of labeled images. Each RGB image $x_{i}$ is paired with a corresponding depth image $d_{i}\in D_{d}$, forming the depth dataset:

(2) $$D_{d}={\left\{d_{i}\right\}}_{i=1}^{M_{x}},$$

where $d_{i}\in X_{d}$. The RGB dataset $D_{x}$ provides rich texture and color information, while the depth dataset $D_{d}$ offers geometric and spatial cues, particularly valuable in architectural environments such as those involving stairs and elevators.

The label space $Y=C^{H\times W}$ shares the spatial dimensions H×W, where C={1,2,3,…,6} corresponds to the six semantic classes: background, upstairs, downstairs, elevator door, elevator frame, and elevator interior.

While *H* and *W* are consistent across images in the dataset for training convenience, the model employs padding and resizing mechanisms to handle variable dimensions during inference. This ensures compatibility across real-world scenarios where input image sizes may differ.

#### 3.2.2. Model Training and Loss Function

The segmentation model *R* is trained to map any input pair of RGB and depth images to the label space *Y*:

(3) $$R:(X,X_{d})\to Y,$$

where $X=R^{H\times W\times 3}$ represents RGB images with spatial dimensions H×W and three channels, and $X_{d}=R^{H\times W\times 1}$ represents depth images with the same spatial dimensions and a single channel.

The dataset is split into training and testing sets using a 7:3 ratio. Training utilizes both RGB and depth inputs to PSPNet with a ResNet-50 backbone, leveraging complementary features from both modalities to improve segmentation accuracy. The model is trained using pixel-wise cross-entropy loss, which measures the divergence between predicted probabilities and ground truth labels. The loss is defined as follows:

(4) $$L_{CE}=-\frac{1}{H\cdot W}\sum_{h=1}^{H}\sum_{w=1}^{W}\sum_{c=1}^{C}y_{h,w,c}\log\left({\hat{y}}_{h,w,c}\right),$$

where $y_{h,w,c}$ represents the ground truth one-hot encoding for class *c* at pixel (h,w):

(5) $$y_{h,w,c}=\left\{\begin{array}{cc} 1, & \mathrm{if}\;\mathrm{class}\;c\;\mathrm{is}\;\mathrm{the}\;\mathrm{ground}\;\mathrm{truth}\;\mathrm{at}\;(h,w),\\ 0, & \mathrm{otherwise}. \end{array}\right.$$

The predicted probabilities ${\hat{y}}_{h,w,c}$ are computed using the softmax function applied to the model’s logits $z_{h,w,c}$, where $z_{h,w,c}$ represents the logarithms of the unnormalized scores for each class rather than probabilities. The softmax function ensures normalization across all classes, transforming these logits into a valid probability distribution. Specifically, the probabilities are calculated as follows:

(6) $${\hat{y}}_{h,w,c}=\frac{\exp(z_{h,w,c})}{\sum_{c^{\prime }}\exp\left(z_{h,w,c^{\prime }}\right)},$$

where the denominator $\sum_{c^{\prime }}\exp\left(z_{h,w,c^{\prime }}\right)$ serves as a normalizing constant, ensuring that the probabilities sum to 1 for each pixel. This guarantees that ${\hat{y}}_{h,w,c}$ is a proper probability distribution over all classes at pixel (h,w).

#### 3.2.3. Skip-Connection Fusion Module

While the pixel-wise cross-entropy loss focuses on independent predictions at each pixel, the SCF module dynamically fuses features from RGB and depth branches through skip-connections at multiple network layers. These skip-connections allow information flow across network layers, preserving spatial and contextual details essential for segmentation.

The SCF module incorporates learnable parameters to optimally weight and combine features from the RGB and depth modalities. This dynamic fusion mechanism ensures that the model effectively captures texture and geometric cues, enhancing segmentation accuracy for complex classes such as elevator frames and interiors. For instance, depth features aid in delineating architectural boundaries, while RGB features provide critical textural information.

Integrated with PSPNet, the SCF module significantly improves segmentation results, particularly in challenging scenarios where ambiguous boundaries or low contrast are prevalent. Future work may involve post-processing techniques such as conditional random fields (CRFs) to further enhance spatial coherence and boundary refinement.

**Figure 1:**
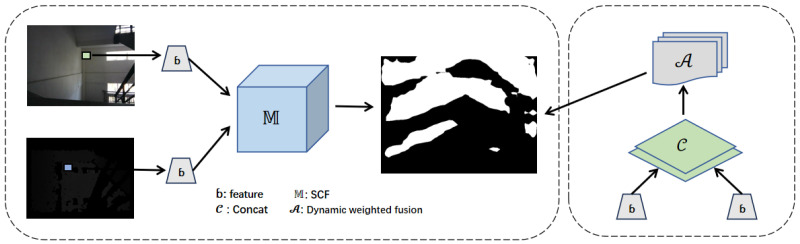
Module flow steps.

As illustrated in  Figure 1, the proposed framework enhances semantic segmentation by effectively integrating depth and RGB features. Initially, feature extraction is performed separately on the RGB and depth images using independent backbone networks. These extracted features are then fused through the proposed Skip-Connection Fusion (SCF) module, which consists of an attention mechanism (dynamic weighting) to adaptively adjust the contribution of each modality and a skip-connection integration to retain fine-grained spatial details. The fused features, enriched with both global context and fine structural information, are subsequently fed into the decoder to generate the final segmentation map. This architecture is particularly effective in recognizing stairs and elevators, which often pose challenges due to occlusion, reflective surfaces, and varying lighting conditions. By leveraging multimodal information, the SCF module significantly improves segmentation accuracy and robustness in complex real-world scenarios.

#### 3.2.4. SCF Module Architecture Design and Placement

The SCF module is designed to address the challenges in semantic segmentation, particularly for complex indoor environments where issues like occlusions, low contrast, and reflective surfaces commonly occur. To enhance segmentation accuracy, the SCF module leverages a dynamic fusion approach, combining RGB and depth features through a learnable weighting mechanism.

The SCF module is placed just before the decoder stage in both the DeepLabv3 and PSPNet architectures. This strategic placement ensures that the fused RGB and depth features are introduced into the model at a point where high-level semantic information is being processed, which allows the model to take advantage of both global and local context during the segmentation process.

The SCF architecture is designed to dynamically adjust the contribution of RGB and depth features by employing a learnable weighting mechanism. This enables the module to adapt to various scenarios more effectively than traditional concatenation methods. By utilizing depth information alongside RGB, the model gains additional geometric and spatial cues that help resolve ambiguities in difficult areas such as staircases and reflective elevator doors.

### 3.3. Feature Fusion via Concatenation

While feature concatenation serves as a straightforward method for RGB–depth fusion, its computational inefficiencies and inability to adapt to contextual significance highlight the need for advanced fusion strategies like SCF. In this section, we first provide an overview of the concatenation approach, followed by an analysis of its computational trade-offs and limitations, which ultimately motivate the introduction of the Skip-Connection Fusion (SCF) module.

#### 3.3.1. Overview of Concatenation

In our methodology, feature concatenation (cat) is employed as an initial approach to fuse RGB and depth feature maps, leveraging their complementary characteristics. Specifically, given the RGB feature map $F_{\mathrm{RGB}}\in R^{H\times W\times C}$ and the depth feature map $F_{\mathrm{Depth}}\in R^{H\times W\times C}$, concatenation produces the fused feature map $F_{\mathrm{Fused}}\in R^{H\times W\times 2C}$. Here, *H* and *W* represent the height and width of the feature maps and *C* denotes the number of channels per feature map. This operation expands the feature dimensionality while preserving spatial consistency.

RGB images $X\in R^{H\times W\times 3}$ encapsulate high-dimensional texture and chromatic information, whereas depth images $X_{d}\in R^{H\times W\times 1}$ convey geometric and structural spatial cues. By concatenating their feature embeddings, a joint representation is formed that combines texture and appearance-based information from RGB features with geometry-based cues from depth features. This fused representation is particularly effective for delineating intricate classes such as upstairs, downstairs, and distinct elevator components.

#### 3.3.2. Computational Trade-Offs

While concatenation offers a straightforward mechanism for integrating features, it introduces notable computational challenges. The expansion of feature dimensionality from *C* to 2C demands additional parameters and memory, increasing the complexity of the network. This leads to longer training times as the model optimizes a larger parameter set to achieve convergence. Furthermore, the larger model size elevates the risk of overfitting, particularly when training data is limited. These factors underline the trade-offs associated with concatenation, where the advantages of retaining modality-specific characteristics come at the cost of increased computational overhead.

#### 3.3.3. Limitations and Transition to SCF

Despite its ability to preserve modality-specific features, naive concatenation has inherent limitations. Treating the concatenated modalities as equally significant disregards their context-specific relevance. For example, RGB features are more effective in visually salient regions like elevator interiors, whereas depth features excel in capturing structural boundaries such as upstairs and downstairs. This uniform weighting hinders the model’s ability to fully leverage the complementary strengths of RGB and depth inputs. Additionally, concatenation does not inherently facilitate cross-modal interaction, limiting the fusion process to a simple aggregation of features without dynamic contextual adaptation.

To address these limitations, we propose the Skip-Connection Fusion (SCF) module, which extends concatenation by incorporating learnable mechanisms for dynamic weighting, refinement, and hierarchical integration of RGB and depth features. The SCF module employs adaptive attention layers, where attention weights are calculated based on spatial and contextual correlations of features from each modality. These weights are then applied to modulate feature contributions, ensuring a balanced and context-aware fusion of RGB and depth information.

Furthermore, the SCF module’s hierarchical integration facilitates robust cross-modal interaction, enabling the extraction of semantically enriched feature representations. These enhancements effectively mitigate the computational inefficiencies introduced by high-dimensional concatenation, offering a more resource-efficient and context-aware approach to feature fusion.

### 3.4. Our Module: Skip-Connection Fusion (SCF)

**Figure 2:**
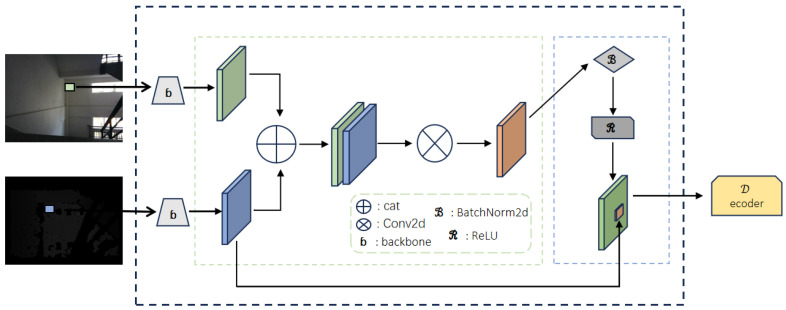
Module detail diagram.

Our segmentation model incorporates the novel Skip-Connection Fusion (SCF) module to fuse RGB and depth features efficiently. The SCF module works as presented in Figure 2.

The Skip-Connection Fusion module integrates RGB and depth features extracted from their respective backbones by concatenating them along the channel dimension, creating a unified representation that combines complementary information from both modalities. This combined feature map undergoes a 1×1 convolution to refine and compress the fused features, followed by batch normalization to stabilize the feature distribution and improve training efficiency. A ReLU activation function is then applied to introduce non-linearity and enhance feature expressiveness. Finally, the processed fused features are added back to the original RGB features using a skip-connection, preserving low-level RGB details while benefiting from the depth-informed enhancements. This design ensures efficient feature integration, maintaining critical spatial and textural details for downstream decoding tasks.

#### 3.4.1. Feature Extraction

We use two separate ResNet backbones to extract features, one for RGB data and one for depth data. Both backbones share a similar architectural structure, leveraging the proven ResNet design for effective feature extraction; however, they are adapted to suit the characteristics of their respective input modalities.

- **RGB Backbone:** This network extracts texture and appearance features from the RGB input $X\in R^{H\times W\times 3}$. It captures rich color information and fine-grained texture details, which are critical for delineating object boundaries and understanding visual context.
- **Depth Backbone:** In contrast, the depth backbone processes the depth input $X_{d}\in R^{H\times W\times 1}$. While it shares the same ResNet architecture, the first convolutional layer is modified to accept single-channel input. This network is optimized to capture geometric and spatial structures, providing complementary cues to the RGB features.

In summary, although both backbones employ similar ResNet architectures for feature extraction, they are tailored to their specific modalities. The RGB backbone focuses on color and texture, while the depth backbone emphasizes geometric information. This complementary design is critical for achieving robust multimodal fusion in challenging segmentation scenarios.

#### 3.4.2. Fusion Process in the SCF Module

To better illustrate the SCF module’s fusion process, we break it down into five key steps:

1. **Feature Extraction:** The input consists of RGB features $F_{\mathrm{rgb}}\in R^{H^{\prime }\times W^{\prime }\times C}$ and depth features $F_{\mathrm{depth}}\in R^{H^{\prime }\times W^{\prime }\times C}$, which are extracted from separate backbone networks trained on RGB and depth images.
  - RGB features capture texture, color, and fine-grained appearance details.
  - Depth features emphasize geometric structure and object boundaries.
2. **Concatenation:** The extracted RGB and depth features are concatenated along the channel dimension:
  (7) $$F_{\mathrm{fused}}=\mathrm{concat}\left(F_{\mathrm{rgb}},F_{\mathrm{depth}}\right),\;F_{\mathrm{fused}}\in R^{H^{\prime }\times W^{\prime }\times 2C}.$$
  This step merges visual and geometric information, allowing the model to leverage complementary cues from both modalities.
3. **Dimensionality Reduction:** To reduce computational complexity and restore the feature channel size to *C*, a 1×1 convolution is applied:
  (8) $$F_{\mathrm{compressed}}={\mathrm{Conv}}_{1\times 1}\left(F_{\mathrm{fused}}\right),\;F_{\mathrm{compressed}}\in R^{H^{\prime }\times W^{\prime }\times C}.$$
  Batch normalization and ReLU activation further refine the output:
  (9) $$F_{\mathrm{processed}}=\mathrm{ReLU}\left(\mathrm{BatchNorm}(F_{\mathrm{compressed}})\right).$$
4. **Attention Mechanism (Dynamic Weighting):** The SCF module dynamically adjusts feature importance using an attention mechanism:
  (10) $$A\left(h,w,c\right)=\sigma \left(f(F_{\mathrm{processed}}\left(h,w,c\right))\right),$$
  where f(·) is a learnable function and σ(·) is an activation function such as Softmax or Sigmoid.
  The computed attention weights are applied to the feature map:
  (11) $$F_{\mathrm{weighted}}\left(h,w,c\right)=A\left(h,w,c\right)\cdot F_{\mathrm{processed}}\left(h,w,c\right).$$
  The attention mechanism consists of the following:
  - **Spatial Attention:** Identifies key regions in the image by emphasizing areas that contribute most to segmentation.
  - **Channel Attention:** Prioritizes informative feature channels, allowing the model to selectively amplify critical depth or RGB features.
  In practice, this dynamic weighting mechanism enables the model to determine when to rely more on depth or RGB based on scene characteristics. For instance, in environments with poor lighting or strong reflections, where RGB features become unreliable, the model assigns higher importance to depth features to ensure accurate segmentation. Conversely, in cases where depth information is noisy or lacks structural details, the RGB features are given more weight to compensate. This adaptive balancing improves the segmentation of challenging structures such as stairs and elevators, where variations in texture, illumination, and perspective often complicate recognition.
5. **Skip-Connection Integration:** A residual connection integrates the refined feature map with the original RGB input:
  (12) $$F_{\mathrm{output}}=F_{\mathrm{rgb}}+F_{\mathrm{weighted}}.$$
  This preserves low-level RGB information while incorporating the enhanced depth features. The skip-connection also improves gradient flow, ensuring stable training and preventing vanishing gradients.

By following these structured steps, summarized in Algorithm 1, the SCF module effectively fuses RGB and depth features, enhancing segmentation performance in complex scenarios.
**Algorithm 1** Skip-Connection Fusion (SCF) module.1:**procedure** Initialize(in_channel)2:      self.in_channel←in_channel3:      self.conv← 1×1 Convolution(input_channels = 2×in_channel, output_channels = in_channel, bias = False) ▹ Use 1×1 convolution to fuse and reduce channels from 2C to *C*4:      self.bn← BatchNormalization(channels = in_channel) ▹ Normalize features for stable training5:      self.relu← ReLU(activation, inplace=True) ▹ Apply non-linearity for better feature learning6:**end procedure**7:**procedure** Forward(rgb, depth)8:      **Feature Concatenation**9:      total←Concatenate(rgb,depth)     ▹ Concatenate RGB and Depth features along the channel dimension$F_{\mathrm{fused}}=\mathrm{concat}\left(F_{\mathrm{rgb}},F_{\mathrm{depth}}\right).$10:      **Feature Compression**11:      fusion←self.conv(total)     ▹ Use 1×1 convolution to compress feature maps$F_{\mathrm{compressed}}={\mathrm{Conv}}_{1\times 1}\left(F_{\mathrm{fused}}\right).$12:      **Feature Normalization**13:      fusion←self.bn(fusion)     ▹ Apply batch normalization to stabilize feature distribution14:      **Non-linearity**15:      fusion←self.relu(fusion)     ▹ Introduce ReLU activation to enhance feature expression16:      **Skip-Connection Integration**17:      output←rgb+fusion     ▹ Fuse the processed features with the original RGB input$F_{\mathrm{output}}=F_{\mathrm{rgb}}+F_{\mathrm{weighted}}.$18:      **return** output19:**end procedure**

#### 3.4.3. Our Dataset

We introduce the Minjiang Dataset, collected from real-world environments at Minjiang University in Fuzhou, Fujian Province, China, covering diverse locations such as teaching buildings, laboratories, and outdoor plazas. To capture varied environmental conditions, a dual-camera setup was employed at two different heights—approximately 0.35 m and 1.05 m—simulating the perspectives of robotic systems. Both RGB and depth data were recorded using an Orbbec Gemini Pro structured-light depth camera, ensuring robust depth perception.

The dataset comprises 2386 images categorized into two primary classes: stairs (1818 images, 76.2%) and elevators (568 images, 23.8%). This distribution reflects real-world architectural layouts, where staircases are more prevalent than elevators. However, the inherent class imbalance in the dataset introduces challenges for accurately segmenting elevator-related classes, which are underrepresented. To mitigate this issue, data augmentation techniques and class-weighted loss functions are employed to enhance the model’s sensitivity to minority classes, thereby reducing bias and promoting more balanced segmentation performance across categories.

Table 2 presents a comparison between our Minjiang Dataset and several widely used RGB-D datasets, including NYU Depth v2 [19], SUN RGB-D [20], and the Stair Dataset with Depth Maps [15].

Compared to NYU Depth v2 and SUN RGB-D, which are general-purpose indoor datasets with a broad range of object classes, the Minjiang Dataset is specifically designed for the semantic segmentation of staircases and elevators—key structural elements in robotic navigation. This targeted focus makes it particularly valuable for real-world applications such as assistive robotics, autonomous indoor navigation, and obstacle avoidance.

Furthermore, while NYU Depth v2 lacks significant perspective variation and the SUN RGB-D dataset contains limited occlusion diversity, our dataset captures images from two different heights (0.35 m and 1.05 m), simulating robotic viewpoints, and incorporates various angles and distances. It also covers diverse lighting conditions (daylight, artificial lighting, and dim environments) and includes occlusions caused by architectural features and reflections, enhancing its robustness in practical scenarios.

In contrast to the Stair Dataset with Depth Maps, which is primarily designed for stair detection, the Minjiang Dataset provides a more comprehensive set of six semantic classes (upstairs, downstairs, elevator door, elevator frame, elevator interior, and background), enabling finer-grained scene understanding.

In summary, the Minjiang Dataset offers the following advantages:

- **Task-specific Design**: Focused on stair and elevator segmentation for robotic perception.
- **Rich Environmental Variation**: Includes varied lighting, occlusions, and camera perspectives.
- **Balanced Complexity and Scale**: Provides a manageable yet diverse dataset for training and benchmarking segmentation models.
- **Fine-grained Annotation**: Supports multi-class segmentation beyond binary stair detection.

Unlike NYU Depth v2 and SUN RGB-D, our dataset focuses specifically on stair and elevator segmentation, making it uniquely valuable for this application.

To improve dataset diversity, images were collected under varied lighting conditions, including natural daylight, artificial indoor lighting, and dim environments. Additionally, multiple viewing angles (ranging from 45° to 90° from the front left and front right) and different camera distances (30–75 cm) were incorporated, ensuring robustness against occlusions, shadows, and reflections.

For segmentation, images were annotated into six distinct classes: upstairs, downstairs, elevator interior, elevator door, elevator frame, and background. These categories focus on key structural elements crucial for robotic navigation and obstacle avoidance.

**Figure 3:**
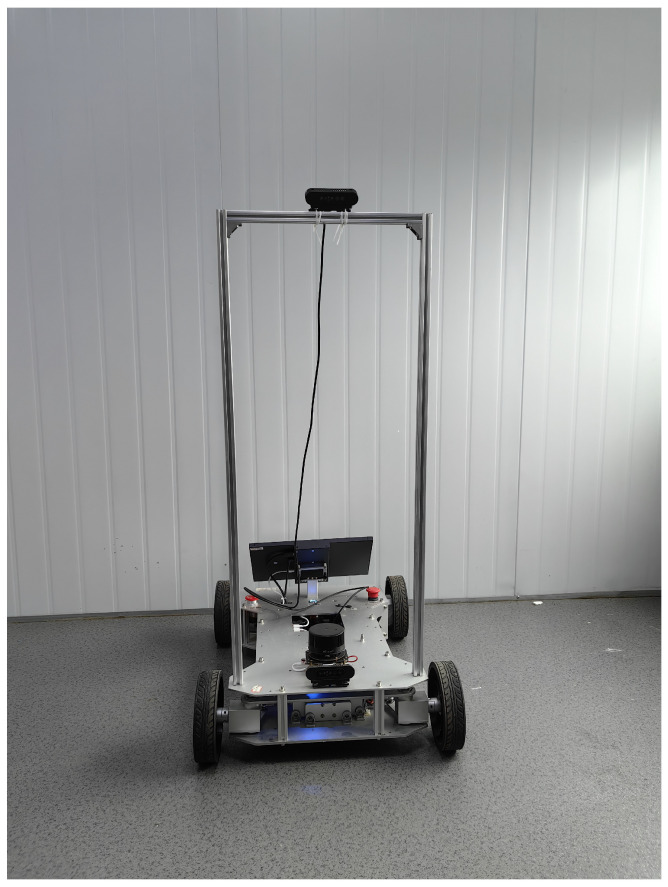
Front side of the robotic platform.

**Figure 4:**
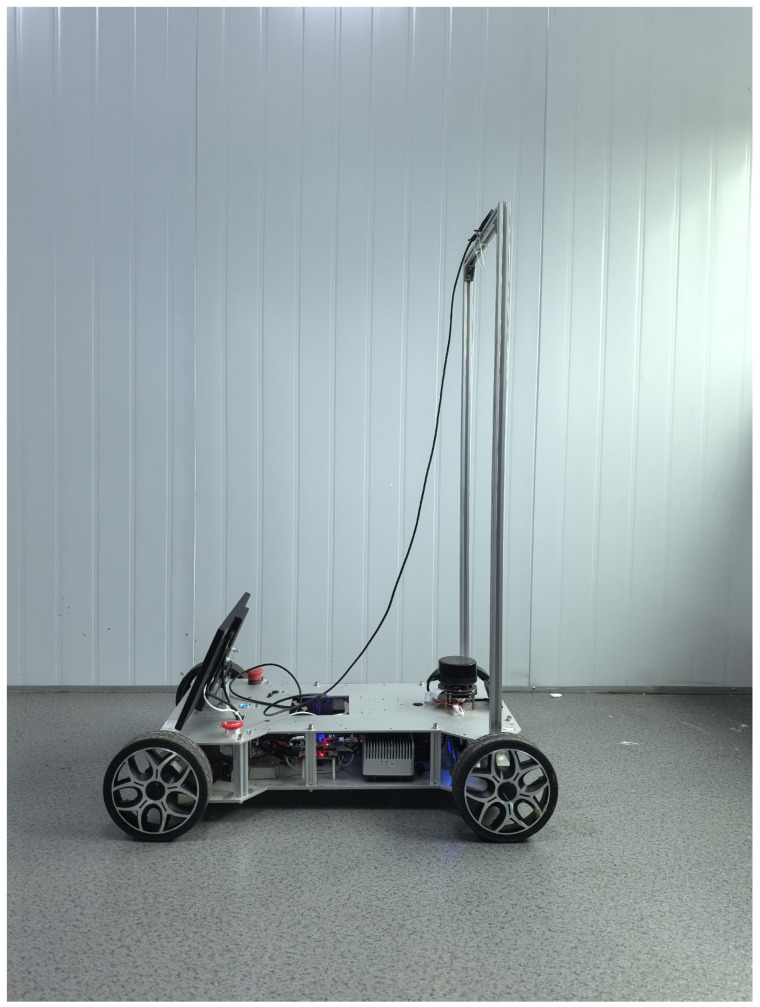
Right side of the robotic platform.

**Figure 5:**
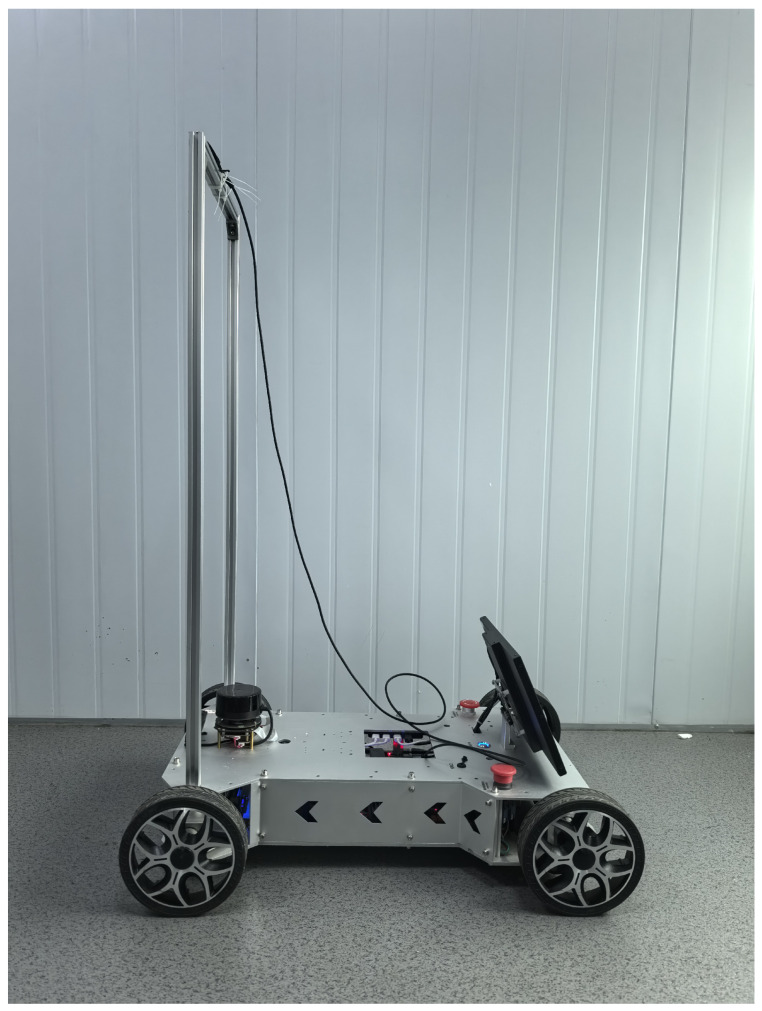
Left side of the robotic platform.

**Figure 6:**
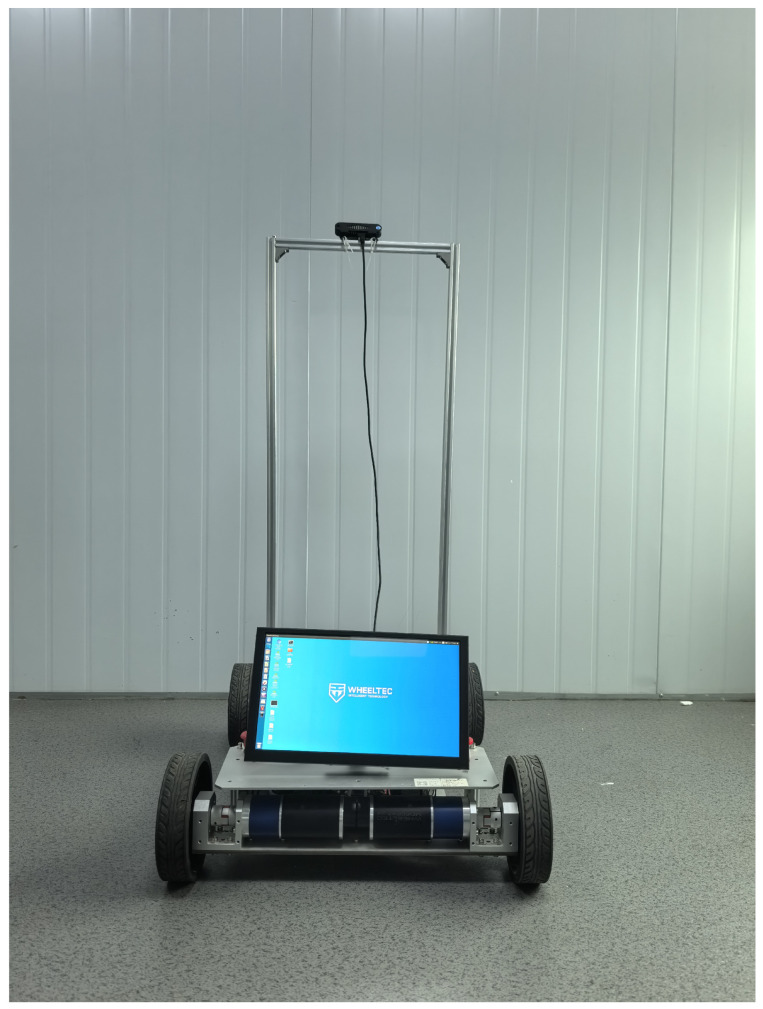
Back side of the robotic platform.

Figure 3, Figure 4, Figure 5 and Figure 6 show the front side, the right side, the left side and the back side of the robotic platform used to collect the Minjiang Dataset, respectively.

All images were pre-processed by segmenting, padding, and resizing to a standardized resolution of 640×480 pixels to maintain consistency and computational efficiency. While resizing may lead to minor loss of fine-grained details—potentially affecting the segmentation of small structures like stair edges or elevator frames—the resolution was chosen as a balance between preserving crucial information and enabling real-time processing.

Despite its diversity in lighting and perspectives, the dataset is relatively small and specific to one university environment, which may limit its generalizability to other settings. Additionally, the dataset contains a higher proportion of stair images, which could introduce a bias favoring stair segmentation. To mitigate these limitations, extensive data augmentation (e.g., random cropping, horizontal flipping, rotation, and brightness adjustments) was applied to increase variability and reduce overfitting. Future work may focus on dataset expansion, domain adaptation, or transfer learning to improve generalization across different architectural environments and real-world scenarios.

## 4. Experimental Evaluation

### 4.1. Experimental Setup

#### 4.1.1. Training Strategy

The model is trained on a workstation equipped with an NVIDIA RTX 3090 GPU, using the PyTorch framework version 2.4. The input size is set to 640×480, and training is conducted for 150 epochs with a batch size of 8. The initial learning rate is 0.001, optimized using Adam without weight decay. Cross-entropy loss (CELoss) is adopted, with ignore_index set to 255 to exclude irrelevant labels.

The model backbone is ResNet-50, configured with an atrous (dilated) convolution strategy of {6,12,18}, replacing strides in the last stage with dilation for better multi-scale feature extraction.

To enhance generalization, we apply data augmentation, including random horizontal flipping (p=0.5), random cropping, and resizing. These augmentations help the model handle variations in scene conditions effectively.

For computational efficiency, we evaluate both training time per epoch and inference time per image. While SCF introduces additional training computation compared to simple RGB-D concatenation, it significantly improves segmentation accuracy with a reasonable increase in inference time, making it suitable for real-time applications.

#### 4.1.2. Evaluation Metric: Mean Intersection over Union (mIoU)

In this study, the primary evaluation metric used to assess the performance of our semantic segmentation model is the mean Intersection over Union (mIoU). mIoU is a standard metric in semantic segmentation tasks and provides a comprehensive measure of the overlap between predicted segmentation and ground truth labels across all classes. The mIoU is defined as follows:

(13) $$\mathrm{mIoU}=\frac{1}{N}\sum_{i=1}^{N}\frac{|P_{i}\cap G_{i}|}{|P_{i}\cup G_{i}|},$$

where

- $P_{i}$: The set of pixels predicted to belong to class *i*;
- $G_{i}$: The set of ground truth pixels for class *i*;
- *N*: The total number of classes.

For our segmentation task, which involves RGB-D fusion to segment architectural structures like stairs and elevators, mIoU effectively quantifies the model’s ability to accurately delineate the boundaries and regions of these classes. This is particularly important as our method integrates RGB and depth information using the Skip-Connection Fusion module and the PSPNet architecture to address challenges such as reflective surfaces, occlusions, and varying lighting conditions.

The choice of mIoU as the evaluation metric over others, such as F1 score or accuracy, is motivated by its alignment with the specific goals of semantic segmentation. Unlike accuracy, which can be skewed by class imbalances, mIoU provides a balanced assessment by explicitly measuring both the true positives and the extent of overlap with false positives and false negatives. Additionally, compared to the F1 score, which focuses on precision and recall for individual classes, mIoU offers a more holistic view of the model’s performance across all classes, making it particularly suitable for multi-class segmentation tasks.

By using mIoU, we can directly compare the performance of our model against other state-of-the-art methods, such as Deeplabv3 and PSPNet, and demonstrate the advantages of our proposed fusion strategy. Specifically, our model’s higher mIoU scores highlight the effectiveness of the learnable skip-connections in fusing complementary features from RGB and depth modalities, as well as the ability to capture global context through pyramid pooling. This metric also underscores the robustness of our method in handling complex scenarios, such as cluttered environments and translucent elevator doors.

The mIoU serves as a critical metric to validate the superiority of our method, ensuring it achieves high segmentation accuracy across all targeted classes while addressing the specific challenges of architectural segmentation.

### 4.2. Comparison with Other Methods

#### 4.2.1. Baseline

Semantic segmentation relies heavily on the quality of feature representation to accurately delineate objects, especially in complex scenarios. As shown in Table 3 and Table 4, baseline methods provide varying degrees of success when applied to challenging segmentation tasks. We present the mIoU for all data, as well as the mIoU in the top 10% of challenging samples.

PSPNet and DeepLabv3 are selected as baseline models due to their effectiveness and widespread adoption in semantic segmentation. PSPNet leverages a pyramid pooling module to capture contextual information at multiple scales, making it well-suited for segmenting complex environments. DeepLabv3 employs atrous spatial pyramid pooling (ASPP) to enhance multi-scale feature extraction, improving performance in scenes with varying object sizes. These models have demonstrated strong performance across diverse datasets and serve as representative benchmarks.

In our experiments, we carefully selected these two models as baselines because of their proven effectiveness in semantic segmentation tasks. PSPNet is well-suited for capturing global context through pyramid pooling, while DeepLabv3 excels at multi-scale feature extraction using atrous convolution. These characteristics are crucial for segmenting complex and cluttered indoor scenes.

However, when applied to our dataset, both models exhibit limitations. PSPNet, while effective in capturing global context, struggles with fine-grained segmentation, particularly in distinguishing small structures such as stair edges and elevator frames. DeepLabv3, despite its ability to handle varying object sizes, is sensitive to depth inconsistencies and reflective surfaces, leading to misclassification in certain challenging scenarios.

In contrast, our SCF-based approach enhances feature fusion between RGB and depth, leading to more accurate segmentation boundaries and improved robustness in real-world conditions. Notably, our model achieves superior performance on the most difficult 10% of samples, demonstrating its ability to handle complex architectural features more effectively than the baseline methods.

The use of SCF enables dynamic weighting between RGB and depth information, which allows the model to better handle challenges like occlusions and reflective surfaces, which both PSPNet and DeepLabv3 struggle with. By incorporating depth features alongside RGB, SCF improves segmentation accuracy, particularly in fine-grained tasks such as detecting stair edges and elevator components.

The RGB-only models offer a foundational benchmark, achieving an mIoU of 88.19% and 85.97% with DeepLabv3 + ResNet-101 and PSPNet + ResNet-50, respectively. However, their inability to capture spatial depth information results in poorer performance on the top 10% most challenging samples (81.69% and 80.49%, respectively). These results indicate that relying solely on RGB features limits the model’s ability to handle ambiguous boundaries and overlapping objects effectively.

Incorporating depth features through direct concatenation improves performance in these challenging cases, with noticeable gains in the top 10% mIoU metric (84.28% for DeepLabv3 and 85.04% for PSPNet). This improvement demonstrates the complementary nature of depth features, which provide geometric and spatial cues absent in RGB data alone. However, the overall mIoU shows negligible or even slightly reduced improvement (88.04% for DeepLabv3 and 86.27% for PSPNet). This limitation arises because simple concatenation lacks a mechanism to dynamically weight or fuse features, thereby failing to fully leverage the depth information.

These findings underscore the need for a more sophisticated fusion strategy capable of dynamically integrating depth features without diluting the contribution of RGB information. The limitations of these baselines justify the introduction of our SCF module, as detailed in the next section.

The SCF (Skip-Connection Fusion) module is specifically designed to address the shortcomings of baseline approaches by dynamically integrating RGB and depth features. The results in Table 3 and Table 4 highlight the significant advantages of using SCF over the baseline methods.

For DeepLabv3 + ResNet-101, the SCF module achieves the highest overall mIoU (88.30%) and top 10% mIoU (85.45%). Similarly, for PSPNet + ResNet-50, the SCF module outperforms the baselines with an mIoU of 86.92% and a top 10% mIoU of 85.72%. These results clearly indicate that the SCF module not only enhances the model’s ability to capture intricate details in challenging samples but also maintains superior performance across the entire dataset.

The dynamic fusion mechanism of SCF allows it to adaptively emphasize the relevant features of both modalities, ensuring a more robust representation of spatial and geometric information. This capability is particularly critical for complex scenarios involving fine-grained classes like upstairs, downstairs, and elevator components, where subtle visual cues must be disambiguated.

One key reason for SCF’s superior performance in the most challenging cases is its ability to handle occlusions and reflections dynamically. Unlike simple concatenation, which treats RGB and depth equally, SCF applies learnable weights that enhance depth features in areas where RGB signals are ambiguous (e.g., reflective floors, glass surfaces, or occluded stair edges). This adaptive fusion allows SCF to preserve fine-grained structural details while suppressing noisy depth information, ultimately leading to more accurate segmentation in difficult scenarios.

In conclusion, the SCF module shows significant improvements over traditional concatenation approaches and RGB-only baselines, establishing itself as a powerful and reliable method for leveraging depth features in semantic segmentation. Its ability to improve both overall and challenging-case performance highlights its potential to address real-world segmentation challenges.

#### 4.2.2. Segmentation Results

In this section, we present some example segmentation results obtained with the proposed SCF method. It is important to note that while concatenating $F_{\mathrm{rgb}}$ and $F_{\mathrm{depth}}$ can increase computational complexity, our module mitigates this through an effective dimensionality reduction mechanism. By dynamically weighting and fusing these features, the module not only reduces computational overhead but also ensures optimal integration of complementary information, thereby enhancing overall performance.

**Figure 7:**
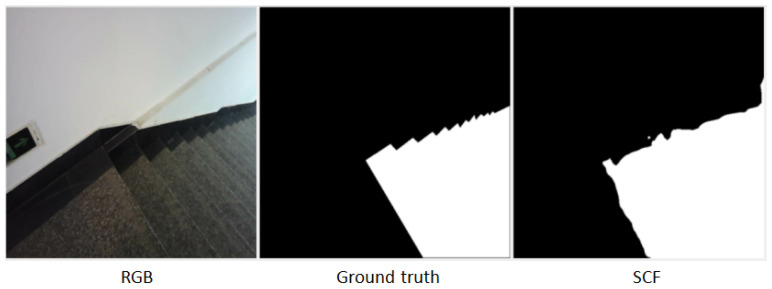
The sample image.

**Figure 8:**
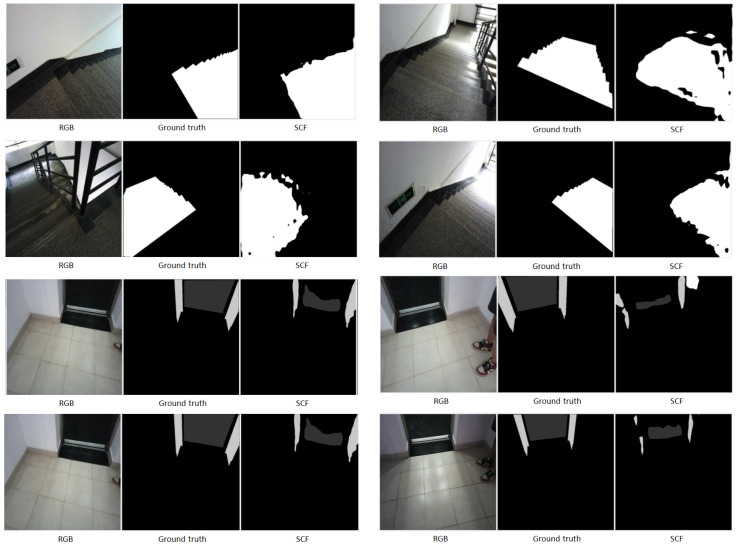
More segmentation results.

The SCF method is particularly robust against challenges such as shadowed staircases or reflective elevator surfaces, as illustrated by the sample image in Figure 7. More segmentation results are shown in Figure 8.

- **Input Image:** Displays the original RGB image used for testing.
- **Ground Truth:** Provides the manually annotated segmentation mask for reference.
- **SCF (Proposed Method):** Highlights improved segmentation accuracy, particularly in ambiguous regions like shadows or low-contrast areas.

#### 4.2.3. SCF vs. Concatenation in Challenging Scenarios

**Figure 9:**
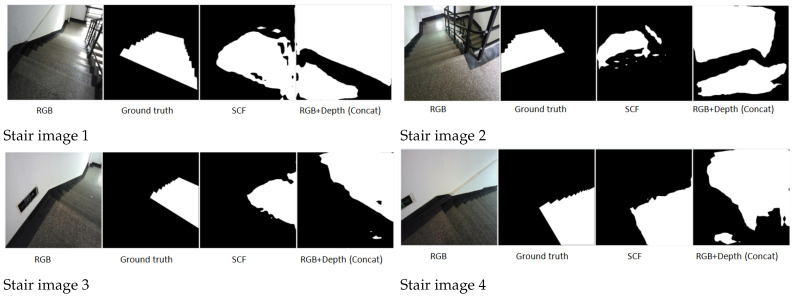
Comparison of SCF and concatenation methods in staircases under strong sunlight. SCF demonstrates better segmentation of reflective and overexposed areas.

**Staircases under Strong Sunlight:** In scenarios where staircases are exposed to strong sunlight, RGB images often suffer from overexposure and reflection, leading to regions with washed-out content and reduced clarity. SCF adaptively leverages depth information to compensate for these deficiencies, resulting in more comprehensive and accurate segmentation. The results are shown in Figure 9, where

- **List 1:** Original RGB images.
- **List 2:** Provides the manually annotated segmentation mask for reference.
- **List 3:** Highlights improved segmentation accuracy, particularly in ambiguous regions like shadows or low-contrast areas.
- **List 4:** Shows partial improvement but struggles with reflective surfaces or overlapping objects.

**Figure 10:**
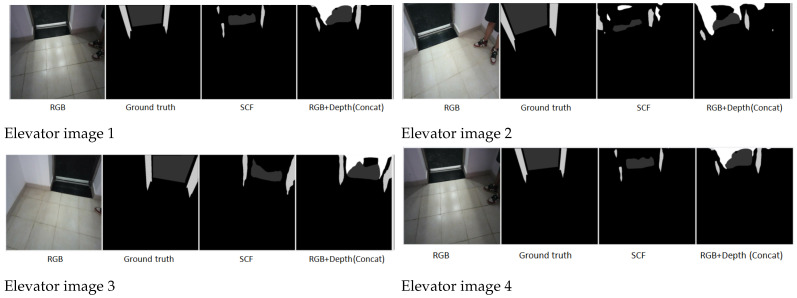
Comparison of SCF and concatenation methods in elevator doors under diffuse reflection. SCF effectively segments reflective surfaces, outperforming the concatenation approach.

**Elevator Doors under Diffuse Reflection:** In addition, we show examples of elevator doors under diffuse reflection in Figure 10.

**Summary:** The qualitative results demonstrate the significant advantages of incorporating depth information through the proposed SCF module. In particular, the SCF-enhanced segmentation process preserves fine-grained structural details of architectural elements such as stairs and elevator frames. The shapes of staircases, railings, and step boundaries are accurately delineated, and elevator interiors and doors—even under challenging conditions like strong lighting, low-contrast shadows, or reflective surfaces—are consistently and precisely segmented.

Unlike methods relying solely on RGB inputs or simple concatenation, which often suffer from blurred boundaries, over-segmentation, or missed details in complex environments, the SCF module adaptively fuses RGB and depth features based on scene characteristics. This ensures robust segmentation performance across diverse lighting conditions and material reflectivities, confirming the SCF module’s effectiveness in improving semantic segmentation for robotic navigation in real-world indoor settings.

#### 4.2.4. Additional Improvements for SCF

##### Proof of SCF Efficacy

**Theoretical Comparison:** The SCF (Skip-Connection Fusion) module is designed to dynamically adjust feature importance through learnable weights, addressing the limitations of simple concatenation methods. In general, the fused feature representation can be computed as follows:

(14) $$F_{\mathrm{SCF}}=\sigma \left(W_{\mathrm{rgb}}\cdot F_{\mathrm{rgb}}+W_{\mathrm{depth}}\cdot F_{\mathrm{depth}}\right)$$

where

- $F_{\mathrm{rgb}}$: Feature map extracted from the RGB modality.
- $F_{\mathrm{depth}}$: Feature map extracted from the depth modality.
- $W_{\mathrm{rgb}}$ and $W_{\mathrm{depth}}$: Learnable weights that dynamically adjust the importance of RGB and depth features, respectively, ensuring robust segmentation. A sensitivity analysis is conducted to evaluate their impact on performance in challenging cases, such as reflective or translucent surfaces.
  Empirical analysis shows that when $W_{\mathrm{rgb}}$ is high $\left(W_{\mathrm{rgb}}\gg W_{\mathrm{depth}}\right)$, segmentation is more sensitive to texture and color but struggles with reflective surfaces, where misleading reflections can cause incorrect classifications. Conversely, when $W_{\mathrm{depth}}$ dominates $\left(W_{\mathrm{depth}}\gg W_{\mathrm{rgb}}\right)$, the model relies more on spatial geometry, improving segmentation in low-texture regions but introducing noise in areas where depth data is unreliable (e.g., glass surfaces).
  To achieve optimal segmentation, we fine-tune these weights dynamically during training using a learnable balancing mechanism:
  (15) $$W_{\mathrm{rgb}}=\frac{e^{\alpha_{\mathrm{rgb}}}}{e^{\alpha_{\mathrm{rgb}}}+e^{\alpha_{\mathrm{depth}}}},\;W_{\mathrm{depth}}=\frac{e^{\alpha_{\mathrm{depth}}}}{e^{\alpha_{\mathrm{rgb}}}+e^{\alpha_{\mathrm{depth}}}},$$
  where $\alpha_{\mathrm{rgb}}$ and $\alpha_{\mathrm{depth}}$ are learnable parameters optimized via backpropagation. This formulation ensures that the model adaptively emphasizes RGB and depth features.
- σ: An activation function, such as *sigmoid* or *softmax*, which normalizes the weighted sum to ensure numerical stability and sparsity.
- $F_{\mathrm{SCF}}$: The final fused feature map, emphasizing key regions for improved performance.

Compared to the simple concatenation method,

(16) $$F_{\mathrm{concat}}=\left[F_{\mathrm{rgb}},F_{\mathrm{depth}}\right]$$

where features are merely stacked along the channel dimension, the SCF module adaptively learns to prioritize relevant features, resulting in more efficient and effective fusion of information across modalities.

**Computational Complexity:** The SCF module introduces additional computational overhead due to the learnable weights and attention mechanisms. The time complexity of SCF is expressed as follows:

(17) $$\mathrm{SCF Time Complexity:}\;O(n_{\mathrm{params}}+n_{\mathrm{fuse}})$$

where

- $n_{\mathrm{params}}$: The number of parameters in the dynamic weighting mechanism.
- $n_{\mathrm{fuse}}$: The computational cost of the feature fusion process.

In contrast, the time complexity of the concatenation method is

(18) $$\mathrm{Concatenation Time Complexity:}\;O(n_{\mathrm{concat}})$$

where $n_{\mathrm{concat}}$ denotes the computational cost of stacking features.

The time and space complexity comparisons are presented in Table 5.

##### Adaptive Attention Mechanism

**Mathematical Explanation:** The SCF module incorporates an adaptive attention mechanism consisting of spatial and channel attention to dynamically emphasize critical features. The spatial attention is computed as follows:

(19) $$A_{\mathrm{spatial}}=\sigma \left(W_{s}\cdot F_{\mathrm{combined}}\right)$$

where

- $W_{s}$: Learnable weight matrix for spatial attention.
- $F_{\mathrm{combined}}$: The combined feature map of RGB and depth modalities.

Similarly, the channel attention is computed as

(20) $$A_{\mathrm{channel}}=\sigma \left(W_{c}\cdot F_{\mathrm{combined}}\right)$$

where $W_{c}$ is the learnable weight vector for channel attention.

The final fused feature map is obtained by combining spatial and channel attention:

(21) $$F_{\mathrm{final}}=A_{\mathrm{spatial}}\cdot A_{\mathrm{channel}}\cdot F_{\mathrm{input}}$$

where

- $F_{\mathrm{input}}$: The input feature map to be refined.
- $F_{\mathrm{final}}$: The final refined feature map.

**Role of Attention Activation (σ):** The activation function σ plays a pivotal role in the SCF module:

- **Dynamic Feature Selection**: By normalizing the attention weights, σ ensures that the model focuses on critical regions while suppressing irrelevant features.
- **Numerical Stability**: Activation functions such as sigmoid or softmax prevent extreme weight values, facilitating stable convergence during training.
- **Sparsity**: Functions like sigmoid introduce sparsity in attention weights, allowing the model to prioritize a smaller subset of features for efficient learning.

By integrating spatial and channel attention with appropriate activation functions, the SCF module enhances its ability to adaptively capture cross-modality interactions and improve segmentation performance, particularly in challenging scenarios.

### 4.3. Ablation Experiment

#### 4.3.1. Objective

The objective of this ablation experiment is to evaluate the individual contributions of different feature fusion strategies to the semantic segmentation performance, specifically within the DeepLabv3 framework. We consider four configurations: using RGB features alone, simple concatenation of RGB and depth features, integration of the proposed Skip-Connection Fusion (SCF) module, and the incorporation of the Cross-Fusion Module (CFM) introduced in CFCG [21].

By comparing overall segmentation accuracy, computational efficiency, and robustness in challenging indoor architectural scenarios, this experiment aims to comprehensively assess the advantages and trade-offs of each fusion strategy. The analysis will demonstrate the effectiveness of SCF relative to both conventional and recent semi-supervised fusion methods, highlighting its suitability for real-time robotic navigation applications.

#### 4.3.2. Experimental Design

Semantic segmentation methods often struggle with performance limitations in challenging scenarios, such as occluded staircases, reflective elevator doors, and low-contrast regions. In this study, we design comparative experiments to evaluate the effectiveness of different feature fusion strategies, including RGB-only, simple concatenation of RGB and depth features, the proposed Skip-Connection Fusion (SCF) module, and the recent Cross-Fusion Module (CFM) introduced in CFCG [21].

These configurations are assessed within the DeepLabv3 framework by comparing segmentation accuracy, computational efficiency, and robustness in typical architectural indoor scenes. The experiments focus on specific challenges, such as accurately segmenting reflective elevator doors, where RGB-only methods often fail due to specular reflections, improving occluded staircase detection by leveraging depth information, and enhancing segmentation in low-contrast regions where RGB images alone lack sufficient detail.

The SCF and CFM modules both address these difficulties by fusing complementary RGB and depth features, but with different fusion mechanisms. SCF applies a learnable skip-connection and adaptive weighting strategy to dynamically emphasize informative regions, while CFM uses a cross-modality guidance approach to refine feature integration. This allows us to comprehensively evaluate the advantages and limitations of each strategy for fine-grained semantic segmentation tasks.

##### Computational Overhead

Both SCF and CFM introduce additional computational complexity compared to simple concatenation, owing to their dynamic feature fusion mechanisms. Specifically, we highlight the following:

- Runtime: SCF performs learnable weight adjustments across feature channels, while CFM involves contour-based feature guidance operations. Both increase runtime compared to concatenation but aim to enhance the integration of RGB and depth features for better segmentation performance.
- Memory Usage: These modules introduce extra parameters and intermediate feature maps, leading to a modest increase in memory consumption. However, this overhead is justified by their ability to improve segmentation accuracy in complex scenes.

Despite these computational demands, both SCF and CFM demonstrate clear performance gains in accuracy and robustness, particularly for difficult indoor environments. The experiments ultimately aim to identify the most suitable fusion strategy that balances segmentation precision with computational efficiency for real-time robotic navigation applications.

#### 4.3.3. Results and Analysis

Table 6 summarizes the performance of the four configurations. The proposed SCF module consistently achieves higher mIoU and improved segmentation accuracy over RGB-only, concatenation, and CFM-based methods, confirming its superior ability to integrate complementary RGB and depth features in complex environments.

##### Key Observations

- RGB-only: Training with only RGB features achieved reasonable accuracy but struggled with challenging scenarios, demonstrating the limitations of single-modality input.
- Concatenation: Simple concatenation of RGB and depth improved top 10% mIoU but introduced marginal degradation in overall mIoU due to suboptimal feature integration.
- CFM: An advanced fusion method, CFM outperformed RGB-only in top 10% mIoU but achieved a lower overall mIoU (85.05%) than concatenation and SCF, indicating its limited robustness in complex indoor scenes.
- SCF (Ours): The SCF module achieved the highest mIoU and top 10% mIoU, demonstrating its ability to dynamically fuse features and enhance segmentation robustness.

##### Significance of SCF Efficacy

The results clearly show that while CFM provides improvements over RGB-only and concatenation in difficult regions, it still falls behind SCF in both overall segmentation accuracy and challenging scenarios. This confirms the efficacy of SCF’s adaptive weighting and skip-connection design for effectively integrating RGB and depth features while mitigating noise, occlusions, and reflections.

##### Computational Overhead

Although SCF introduced an increase in runtime compared to RGB-only, it offered superior segmentation accuracy relative to both concatenation and CFM. Notably, CFM also incurred an increase in runtime but delivered a lower mIoU than SCF, further validating SCF’s balanced trade-off between performance and computational cost.

In conclusion, these experiments comprehensively validate the proposed SCF module’s advantages over both baseline and advanced feature fusion methods, reinforcing its value for robust semantic segmentation in real-world indoor robot navigation.

## 5. Conclusions and Future Work

In this paper, we proposed the Skip-Connection Fusion (SCF) module to enhance RGB-D semantic segmentation by effectively integrating depth information with RGB features. The experimental results demonstrate that SCF exhibits superior adaptability in challenging scenarios, achieving a 5.23% improvement, particularly in cases with strong sunlight, diffuse reflections, and reflective surfaces. By leveraging depth cues, SCF compensates for the deficiencies of RGB inputs, significantly improving segmentation accuracy in these difficult conditions.

**Limitations:**

- **Handling Highly Dynamic Scenes:** Although our SCF module improves segmentation in challenging static architectural scenes, it may face difficulties when dealing with highly dynamic or rapidly changing environments, which requires further adaptation.
- **Computational Cost in Edge Cases:** While the proposed method achieves a good balance between accuracy and efficiency, certain complex scenes with extreme occlusions or reflections can still increase computational overhead.
- **Generalization to Other Architectural Elements:** Our approach is specifically optimized for stairs and elevators; its effectiveness on other indoor architectural components or outdoor environments needs further investigation.

**Strengths:**

- **Adaptive Fusion of RGB and Depth:** The learnable Skip-Connection Fusion (SCF) module dynamically balances complementary modalities, improving robustness against occlusion, reflection, and lighting variations that commonly challenge traditional methods.
- **Integration with Contextual Features:** By leveraging PSPNet’s pyramid pooling, our framework captures rich global and local context, significantly enhancing segmentation accuracy in cluttered and complex indoor scenes.
- **Task-Specific Optimization:** Tailoring the model specifically for stairs and elevators allows achieving superior segmentation performance without compromising real-time computational requirements, crucial for robotic navigation.
- **Comprehensive Dataset and Evaluation:** The newly collected, diverse real-world dataset simulating robot vision provides a solid benchmark to validate the effectiveness and generalization capability of the proposed approach.
- **Practical Impact:** The improved segmentation precision contributes directly to safer and more reliable robot navigation in multi-floor architectural environments, addressing a critical real-world need often overlooked in generic segmentation models.

Quantitative and qualitative comparisons validate that SCF consistently outperforms standard feature concatenation in terms of mean Intersection over Union (mIoU). The improved performance underscores the effectiveness of our adaptive feature fusion strategy, highlighting its potential for real-world applications in robotic perception and autonomous navigation.

While our method demonstrates strong segmentation performance, there remain areas for future improvement. One potential direction is enhancing robustness in dynamic environments where lighting conditions and object appearances change over time. Further, improving the generalizability of SCF to different architectural layouts beyond the current dataset will be crucial for broader deployment. Incorporating additional sensor modalities, such as LiDAR or thermal imaging, could also enrich scene understanding by providing complementary spatial and material information.

For future work, we aim to refine the SCF module by integrating more sophisticated attention mechanisms to further optimize feature selection and fusion. Additionally, optimizing computational efficiency will be a key focus to ensure real-time deployment in embedded and edge computing environments. Expanding the dataset to encompass more diverse indoor and outdoor settings, such as escalators, ramps, and automated doors, will also be considered, allowing the model to better adapt to real-world variations. Finally, exploring post-processing techniques such as conditional random fields (CRFs) or transformer-based refinements could further improve segmentation consistency and fine-grained details, making SCF more suitable for safety-critical applications in robotics and assistive navigation.

## Figures and Tables

**Table 1:** Comparison of related methods.

Method	Main Idea	Advantages	Limitations
FuseNet	Dual-stream network fuses RGB and depth in the decoding stage	Improves segmentation accuracy; simple and easy to implement	Direct fusion, lacks adaptive adjustment; sensitive to depth noise
RDF-GAN	Uses GAN for RGB-D semantic segmentation	Enhances feature representation; suitable for complex scenes	Hard to train, prone to mode collapse; high computational cost
Our Method	Uses SCF for RGB-D fusion with PSPNet	Learnable fusion improves complementarity; PSPNet enhances accuracy	Generalization needs further validation; more fusion comparisons needed

**Table 2:** Dataset description and comparison.

Dataset	Image Count	Classes	Lighting Conditions	Occlusions	Perspective Variation
Minjiang Dataset (Ours)	2386	6	High	Yes	Yes
NYU Depth v2 [19]	1449	13	Moderate	Yes	No
SUN RGB-D [19]	10,335	37	High	Limited	Yes
Stair Dataset with Depth Maps	2996	2	High	Yes	Yes

**Table 3:** Performance of DeepLabv3 + ResNet-101 in mIoU. Best results are shown in bold.

DeepLabv3 + ResNet-101			
**Model**	**mIoU (%)**	**Top 10% mIoU (%)**	**Improvement**
Only RGB	88.19	81.69	Baseline
Depth + RGB (Concat)	88.04	84.28	+2.59% (Top 10%)
With SCF	88.30	85.45	+3.76% (Top 10%)

**Table 4:** Performance of PSPNet + ResNet-50 in mIoU. Best results are shown in bold.

PSPNet + ResNet-50			
**Model**	**mIoU (%)**	**Top 10% mIoU (%)**	**Improvement**
Only RGB	85.97	80.49	Baseline
Depth + RGB (Concat)	86.27	85.04	+4.55% (Top 10%)
With SCF	86.92	85.72	+5.23% (Top 10%)

**Table 5:** Time and space complexity comparisons.

Method	Theoretical Time Complexity	CPU Time (ms)	Space Complexity
Concatenation	$O(n_{\mathrm{concat}})$	4784	O(m)
SCF	$O(n_{\mathrm{params}}+n_{\mathrm{fuse}})$	3749	O(m+δ)

Here, *m* is the memory required for storing concatenated features and δ is the additional memory required for SCF’s learnable parameters.

**Table 6:** Ablation study results: comparison of segmentation performance (mIoU and top 10% mIoU) and computational overhead for different fusion strategies in DeepLabv3 + ResNet-101. Best results are shown in bold.

Method	mIoU (%)	Top 10% mIoU (%)	Normalized Runtime Increase
RGB-only	88.19	81.69	0
Concatenation	88.04	84.28	+100%
CFM [21]	85.05	83.82	+270%
SCF (Ours)	**88.30**	**85.45**	+78%

## Data Availability

The dataset is available on https://github.com/ZZZHHU/MinJiang-Dataset/tree/master (accessed on 20 July 2025).
